# Circulating hematopoietic stem cells and putative intestinal stem cells in coeliac disease

**DOI:** 10.1186/s12967-015-0591-0

**Published:** 2015-07-11

**Authors:** Anna Chiara Piscaglia, Sergio Rutella, Lucrezia Laterza, Valentina Cesario, Mariachiara Campanale, Immacolata Alessia Cazzato, Gianluca Ianiro, Federico Barbaro, Luca Di Maurizio, Giuseppina Bonanno, Tonia Cenci, Giovanni Cammarota, Luigi Maria Larocca, Antonio Gasbarrini

**Affiliations:** Endoscopy and Gastroenterology Unit, State Hospital, Borgo Maggiore, Republic of San Marino; Institute of Internal Medicine and Gastroenterology, “A. Gemelli” Hospital, Catholic University, Rome, Italy; Division of Translational Medicine, Clinical Research Centre, Sidra Medical and Research Centre, PO Box 26999, Burj Doha, 8th Floor, Doha, Qatar; Endoscopy and Gastroenterology Unit, “S. Caterina Novella” Hospital, Galatina, Lecce Italy; Institute of Gynecology, “A. Gemelli” Hospital, Catholic University, Rome, Italy; Institute of Pathology, “A. Gemelli” Hospital, Catholic University, Rome, Italy

**Keywords:** Stem cells, Coeliac disease, Intestinal stem cells, Mucosal repair, CD34, CD133, Lgr5

## Abstract

**Background:**

The intestinal stem cells (ISC) modulation and the role of circulating hematopoietic stem cells (HSC) in coeliac disease (CD) are poorly understood. Our aim was to investigate the longitudinal modifications in peripheral blood HSC traffic and putative ISC density induced by gluten-free diet (GFD) in CD.

**Methods:**

Thirty-one CD patients and 7 controls were enrolled. Circulating CD133^+^ and CD34^+^ HSC were measured by flow cytometry, at enrolment and after 7 days and 1, 3, 6, 12, and 24 months of GFD. Endoscopy was performed at diagnosis and repeated at 6, 12, and 24 months following GFD. We used the Marsh-Oberhuber score to evaluate the histological severity of duodenal damage; immunohistochemistry was employed to measure the intraepithelial lymphoid infiltrate (IEL, CD3^+^ lymphoid cells) and the putative ISC compartment (CD133^+^ and Lgr5^+^ epithelial cells).

**Results:**

At enrolment, circulating HSCs were significantly increased in CD patients and they further augmented during the first week of GFD, but progressively decreased afterwards. CD patients presented with villous atrophy, abundant IEL and rare ISC residing at the crypt base. Upon GFD, IEL progressively decreased, while ISC density increased, peaking at 12 months. After 24 months of GFD, all patients were asymptomatic and their duodenal mucosa was macroscopically and histologically normal.

**Conclusions:**

In active CD patients, the ISC niche is depleted and there is an increased traffic of circulating HSC versus non-coeliac subjects. GFD induces a precocious mobilization of circulating HSC, which is followed by the expansion of the local ISC compartment, leading to mucosal healing and clinical remission.

## Background

Coeliac disease (CD) is a chronic inflammatory disease of the small bowel, triggered by dietary gluten, in genetically susceptible individuals [1]. The diagnosis of CD is based on the presence of characteristic lesions in small intestinal biopsy samples (intraepithelial lymphocytosis, elongation of the crypts and villous atrophy). However, since villous atrophy is not specific, CD diagnosis also requires a positive serology for anti-tissue-transglutaminase (atTG) antibodies or anti-endomysial antibodies (EMA), and it is confirmed by clinical and/or histological remission following gluten removal from diet [2]. The clinical spectrum of CD is very wide: patients can be asymptomatic (*subclinical*), present with trivial or apparently unrelated symptoms (*non*-*classical CD*), or be severely symptomatic, with frank malabsorption symptoms (*classical CD*) [3]. Moreover, CD patients might present with symptoms of associated autoimmune diseases, such as type 1 diabetes and autoimmune thyroiditis [4]. CD treatment is based solely on dietary intervention, the so-called gluten-free diet (GFD). With maintenance of a GFD, symptoms and serum coeliac antibodies gradually disappear, and healing of the intestinal damage typically occurs within 24 months of diet [1].

Despite the pathogenic interactions among genetic, immunological, and environmental factors in CD have been extensively investigated, little is known about intestinal stem cell (ISC) modulation and deregulation during the disease course and about the possible contribution of circulating bone marrow-derived stem cells to intestinal regeneration upon the commencement of a GFD. Aim of this study was to assess the circulating hematopoietic stem cells (HSC) traffic and putative ISC (CD133^+^/Lgr5^+^ crypt cell) density in active CD patients and to investigate their longitudinal modifications induced by GFD.

## Methods

### Patients and controls

Patients were enrolled at the time of CD diagnosis, which was based upon clinical and serologic findings and subsequently confirmed by histology. After the commencement of a GFD, dietary compliance was established based on dietary history and negativization of EMA and atTG.

The control population consisted of non-coeliac subjects, randomly selected from the same geographical area of CD patients, with no family history of CD or other autoimmune diseases, undergoing an upper endoscopy to investigate dyspepsia and/or heartburn or for positive family history for gastric cancer and expressing their will to participate to the study before the exam. Controls were finally enrolled if their esophageal-gastro-duodenoscopies resulted negative for coeliac, neoplastic, or inflammatory diseases.

Patients and controls were recruited at the Endoscopy Unit of the Department of Internal Medicine, “A. Gemelli” Hospital, Catholic University of Rome. A written informed consent was obtained from all participants before entering the study protocol. The protocol and the consent form had been approved by the local Ethical Committee and conformed to the ethical guidelines of the Declaration of Helsinki (1975).

### Collection of peripheral blood samples and flow cytometry

At baseline, 5 ml of peripheral blood were collected from eligible patients and controls. Blood samples from a subset of CD patients were also collected 7 days and 1, 3, 6, 12, and 24 months after the beginning of the GFD.

All the specimens were collected in heparinized tubes and immediately prepared for analysis. The count of CD133^+^ and CD34^+^ cells co-expressing CD45 was measured by conventional dual-color flow cytometry, using phycoerythrin (PE)-conjugated anti-CD133/2 antibodies (Miltenyi Biotec, Bergisch Gladbach, Germany) or PE-conjugated anti-CD34 antibodies [BD Biosciences (BD), Mountain View, CA, USA], in combination with fluorescein isothiocyanate (FITC)-conjugated anti-CD45 antibodies (BD). Fluorochrome-conjugated isotype-matched monoclonal antibodies were used to establish background fluorescence. Briefly, small aliquots of peripheral blood were incubated with the above-mentioned antibodies for 30 min at 4°C. After red cell lysis with the FACS lysing solution (BD), the samples were washed twice in PBS (Sigma, St Louis, MO, USA) and kept at 4°C until analysis. Samples were acquired on a FACS-Calibur^®^ flow cytometer (BD): at least 5 × 10^4^ events/determination were acquired in list mode and the results were analyzed using the Cell Quest^®^ software (BD).

The percentage of positive cells among white blood cells (WBC) was calculated by subtracting the value of the appropriate isotype controls. All the data shown in the present report refer to the fraction of CD133^+^/CD45^+^ and CD34^+^/CD45^+^ cells that are representative of immature hematopoietic cells.

### Upper gastrointestinal endoscopy, duodenal sample collection, atTG and EMA serology

Upper gastrointestinal endoscopy with duodenal biopsies (6 specimens from the distal duodenum) was performed on patients with positive serology for atTG IgA and EMA. Once CD diagnosis was histologically confirmed, the patients started a GFD; during the follow-up, they underwent repeated endoscopies with collection of duodenal biopsies at 6, 12, and 24 months following the beginning of the GFD. At the same time points, patients were clinically evaluated and serum levels of atTG and EMA were measured.

Control subjects underwent a single upper gastrointestinal endoscopy with duodenal biopsies (6 specimens from the distal duodenum); at enrollment, they were also clinically evaluated and serum levels of atTG and EMA were measured.

### Histology and immunohistochemistry

Histological severity of duodenal damage was evaluated according to the Marsh classification modified by Oberhuber [5] on 4-µm paraffin sections stained with hematoxylin-eosin (H&E): infiltrative lesions with intraepithelial lymphocytosis were defined as Marsh I, infiltrative/hyperplastic lesions as Marsh II, and partial, subtotal, and total villous atrophy as Marsh III (a, b and c, respectively).

Lymphoid infiltrate (CD3) and putative ISC density (CD133/Lgr5) were assessed by immunohistochemistry on 4-µm paraffin sections. Immunohistochemistry was performed on deparaffinized sections using the avidin–biotin-peroxidase complex method (ABC-Elite Kit; Vector Laboratories, Burlingame, CA, USA). For antigen retrieval, paraffin sections were microwave-treated in 0.01 M citric acid buffer, pH 6.0 (2 cycles for 5 min each at 750 W), followed by inhibition of endogenous peroxidase with 3% H_2_O_2_ for 5 min. Then, the sections were incubated with the following primary antibodies: anti-CD3 (rabbit polyclonal; dilution 1:50; Dako; Glostrup, Denmark); CD133 (rabbit polyclonal; dilution 1:50; Biocare Medical, Concord, CA) and anti-Lgr5 (anti-G Protein-Coupled Receptor GPR49, rabbit polyclonal, dilution 1:1,000; MBL Int. Corp., Woburn, MA, USA). After a 1-h incubation at room temperature, immunodetection was performed using goat anti-rabbit secondary antibody (Vector Laboratories) and freshly made diaminobenzidine as a chromogen.

The number of intraepithelial lymphocytes (IEL) and epithelial cell nuclei in a randomly chosen, uninterrupted length of surface (villous) epithelium (>500 cells) was counted and the mean for the number of IEL/100 cells was calculated.

Quantification of ISC was obtained through the count of the number of Lgr5^+^CD133^+^ epithelial cells per crypt from five randomly chosen crypts and the computation of the mean value/crypt.

The entire area of the epithelium in each tissue section was measured on light microscopy using ×20 objective lenses and ×10 eyepiece.

### Statistical analysis

Regarding the circulating CD133^+^ and CD34^+^ HSC counts, the results were expressed as mean ± SD. Student’s t test for unpaired data was used for the statistical analysis.

As for the assessment of villous atrophy, lymphoid infiltrate and intestinal CD133^+^ and Lgr5^+^ cells, statistical analysis was performed using the Mann–Whitney U test.

A *p* value <0.05 was considered statistically significant. The data analysis was generated using Microsoft Excel software (Microsoft Corporation, Redmond, WA, USA) and Real Statistics Resource Pack software (Release 3.5; Copyright 2013–2015 Charles Zaiontz; http://www.real-statistics.com).

## Results

### Patients and controls

Thirty-one consecutive coeliac patients were enrolled from January 2008 to June 2009 (median age 39 years; F-to-M ratio = 2.1). Most patients showed minor symptoms (i.e., anemia, altered bowel habits, abdominal pain and bloating) or symptoms related to associated diseases (i.e., thyroiditis, diabetes). All patients presented with high titer of serum atTG and EMA; upper endoscopy showed in most cases the disappearance or the reduction of Kerckring folds, associated with absence or hypotrophy of the villi at the water immersion technique [6]. Histological examination of intestinal biopsies confirmed the presence of sub-total or total villous atrophy and IEL >25/100 (Marsh III) in all subjects. Patients’ baseline characteristics are enlisted in Table 1.

**Table 1:** Patients’ baseline characteristics

Pt	Sex	Age	Ab	Gastrointestinal symptoms	Other symptoms	Marsh-Oberhuber
#1	M	34	Pos	Diarrhea	Dermatitis herpetiformis, anemia	IIIc
#2	F	29	Pos	Constipation, abdominal bloating	None	IIIc
#3	M	63	Pos	Anemia, dyspepsia	None	IIIb
#4	M	22	Pos	Hypertransaminasemia, diarrhea	None	IIIc
#5	F	31	Pos	None	Type 1 diabetes	IIIc
#6	M	64	Pos	Family history of CD	None	IIIc
#7	F	57	Pos	None	Primary biliary cirrhosis, anemia	IIIc
#8	F	24	Pos	None	Anemia	IIIb
#9	F	46	Pos	Diarrhea, weight loss, malabsorption	Osteoporosis, anemia	IIIc
#10	F	21	Pos	Abdominal pain, constipation	Anemia	IIIc
#11	F	70	Pos	Diarrhea, abdominal bloating	Anemia	IIIb
#12	F	40	Pos	Abdominal discomfort	Thyroiditis	IIIb
#13	F	50	Pos	Constipation, abdominal bloating	Thyroiditis	IIIa
#14	F	50	Pos	Abdominal pain, diarrhea	Anemia	IIIa
#15	M	38	Pos	Abdominal bloating	Osteoporosis, anemia	IIIc
#16	M	32	Pos	Diarrhea	None	IIIb
#17	M	49	Pos	Abdominal pain and bloating	None	IIIb
#18	F	23	Pos	Occasionally altered bowel habits	None	IIIc
#19	M	26	Pos	Diarrhea	None	IIIc
#20	F	30	Pos	Diarrhea	Anemia	IIIb
#21	F	42	Pos	Constipation, abdominal bloating	Anemia	IIIc
#22	F	43	Pos	Family history of CD	None	IIIa
#23	F	23	Pos	None	Fatigue	IIIc
#24	F	21	Pos	Abdominal bloating	Rash	IIIa
#25	F	28	Pos	Dyspepsia	Fatigue, anemia	IIIc
#26	M	45	Pos	None	Infertility	IIIb
#27	F	46	Pos	None	Anemia	IIIc
#28	F	39	Pos	Weight loss	Dermatitis herpetiformis, anemia	IIIc
#29	F	25	Pos	Diarrhea	Thyroiditis	IIIb
#30	F	46	Pos	Abdominal pain, altered bowel habits	Type 1 diabetes	IIIc
#31	M	55	Pos	Diarrhea	None	IIIc

*Pts* patients, *M* male, *F* female, *Ab* antibodies (atTG and/or EMA), *pos* positive, *CD* coeliac disease.

A total of 9 controls undergoing upper endoscopy for dyspepsia, heartburn, or family history of gastric cancer, matched for sex and age (median age 40 years; F:M ratio = 2) were enrolled immediately before the endoscopic examination. Two subjects were subsequently excluded from the study, because they had mild gastritis at endoscopy. In the remaining 7 individuals (average age 38 years; F-to-M ratio = 2.5), in whom there were no pathological endoscopic findings, biopsies were taken from the inferior duodenum for histological examination. Controls’ baseline characteristics are enlisted in Table 2.

**Table 2:** Controls’ baseline characteristics

Ctrls	Sex	Age	Ab	Gastrointestinal symptoms	Marsh-Oberhuber
#1	F	43	Neg	Heartburn	0
#2	F	38	Neg	Dyspepsia	0
#3	F	61	Neg	Family history of gastric cancer	0
#4	M	23	Neg	Dyspepsia, epigastric pain	0
#5	F	38	Neg	Family history of gastric cancer	0
#6	M	21	Neg	Heartburn	0
#7	F	44	Neg	Dyspepsia	0

*Ctrls* controls, *M* male, *F* female, *Ab* antibodies (atTG and/or EMA), *neg* negative.

### Longitudinal modifications of peripheral blood HSC traffic in coeliac patients following a gluten-free diet

WBC count of all patients and controls was within the normal range (4.1–9.8 × 10^9^/L) at enrolment. The absolute number and percentage of both circulating CD133^+^CD45^+^ and CD34^+^CD45^+^ cells were significantly higher in coeliac patients versus controls, with no differences observed when comparing male and female patients (data not shown). In particular, circulating CD34^+^ and CD133^+^ cell percentage in controls ranged from 0.011 to 0.059% (0.028 ± 0.020%) and from 0.008 to 0.019% (0.011 ± 0.004%), respectively, whereas in CD patients, average percentage was 0.166 ± 0.63% for CD34^+^ cells (*p* < 0.05) and 0.020 ± 0.006% for CD133^+^ cells (*p* < 0.05).

**Figure 1:**
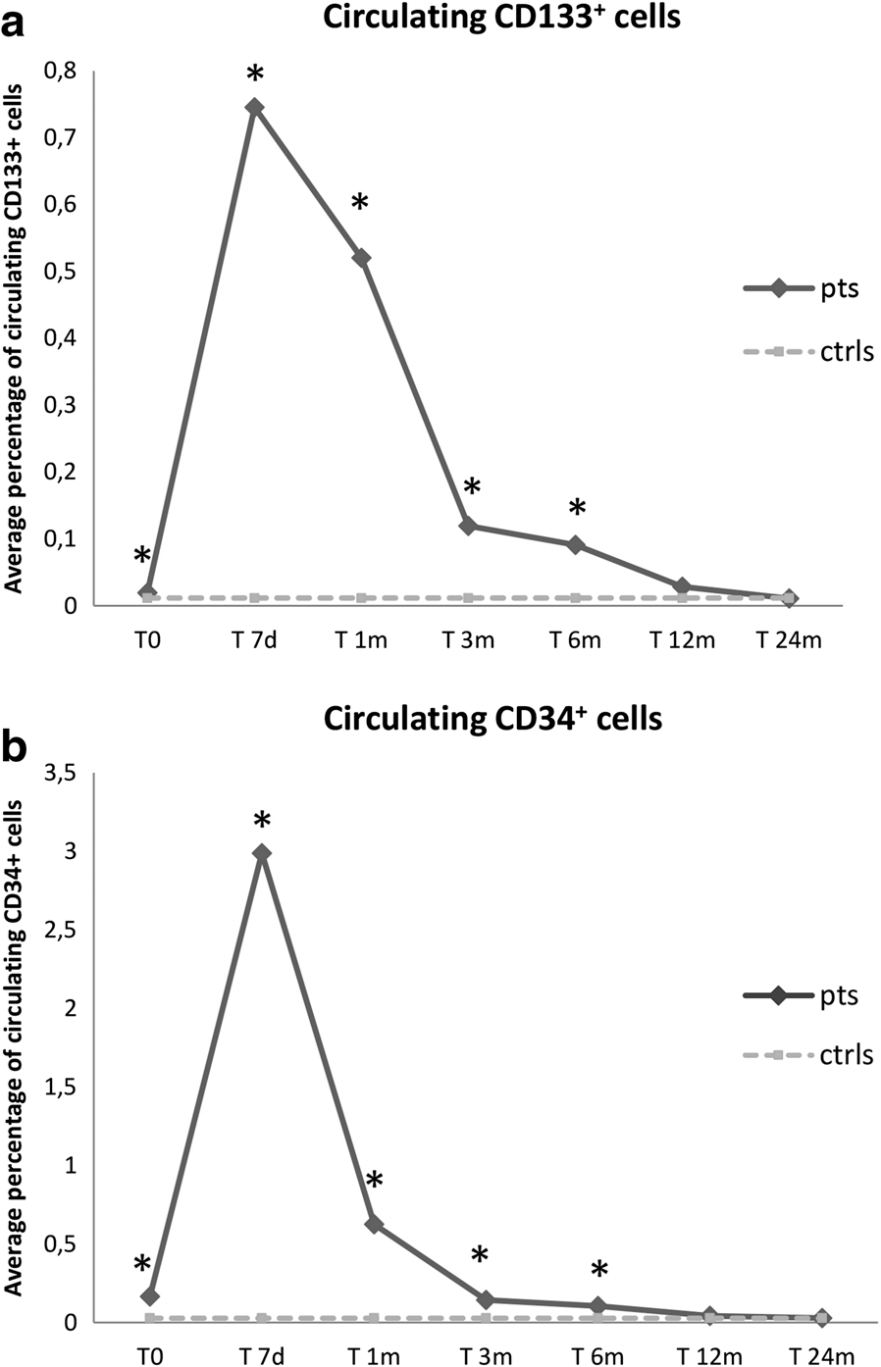
Longitudinal modifications of circulating HSC traffic in CD patients upon starting dietary intervention. **a** Average percentage of circulating CD133^+^ cells at baseline (T0) and at various time-points following gluten removal from diet. **b** Average percentage of circulating CD34^+^ cells at baseline (T0) and at various time-points following gluten removal from diet. *pts* patients, *ctrs* controls, *T0* baseline, *T7d* 7 days following gluten removal from diet, *T1m, T3m, T6m, T12m and T24m* 1, 3, 6, 12 and 24 months following gluten removal from diet, respectively. *p < 0.05.

Following 7 days of GFD, a significant increase of circulating HSC traffic was observed in CD patients (mean ± SD CD133^+^ cells 0.745 ± 0.125%; mean ± SD CD34^+^ cells 2.999 ± 0.406%, *p* < 0.05 versus baseline values). Afterwards, PB HSC cells progressively decreased until reaching the same values as the controls after 12 months of GFD. Figure 1 depicts the longitudinal modifications of circulating HSC traffic in CD patients upon starting dietary intervention. Table 3 enlists the average and standard deviation of CD133^+^ and CD34^+^ cell percentages in GFD-treated coeliac patients during the follow-up, and the relative *p* values of the comparison with controls and active CD patients at the various time-points.

**Table 3:** Circulating CD133^+^ and CD34^+^ HSC in GFD-treated coeliac patients during follow-up

Timing	T0		T7d		T1m		T3m		T6m		T12m		T24m	
HSC	CD133	CD34	CD133	CD34	CD133	CD34	CD133	CD34	CD133	CD34	CD133	CD34	CD133	CD34
Mean %	0.020	0.166	0.745	2.988	0.520	0.626	0.120	0.143	0.091	0.106	0.028	0.042	0.011	0.029
SD %	0.006	0.063	0.125	0.407	0.092	0.093	0.047	0.019	0.024	0.017	0.020	0.008	0.004	0.012
*p* value versus ctrls	<0.05	<0.05	<0.05	<0.05	<0.05	<0.05	<0.05	<0.05	<0.05	<0.05	NS	NS	NS	NS
*p* value versus T0 CD			<0.05	<0.05	<0.05	<0.05	<0.05	NS	<0.05	<0.05	NS	<0.05	<0.05	<0.05

The last two rows represent the *p* values of the comparison between controls and active CD patients at the various time-points.

*SD* standard deviation, *T0* baseline, *T7* 7 days following gluten removal from diet, *T1m, T3m, T6m, T12m and T24m* 1, 3, 6, 12 and 24 months following gluten removal from diet, respectively, *NS* non-significant.

### Longitudinal modifications of putative intestinal stem cell density in coeliac patients following a gluten-free diet

**Figure 2:**
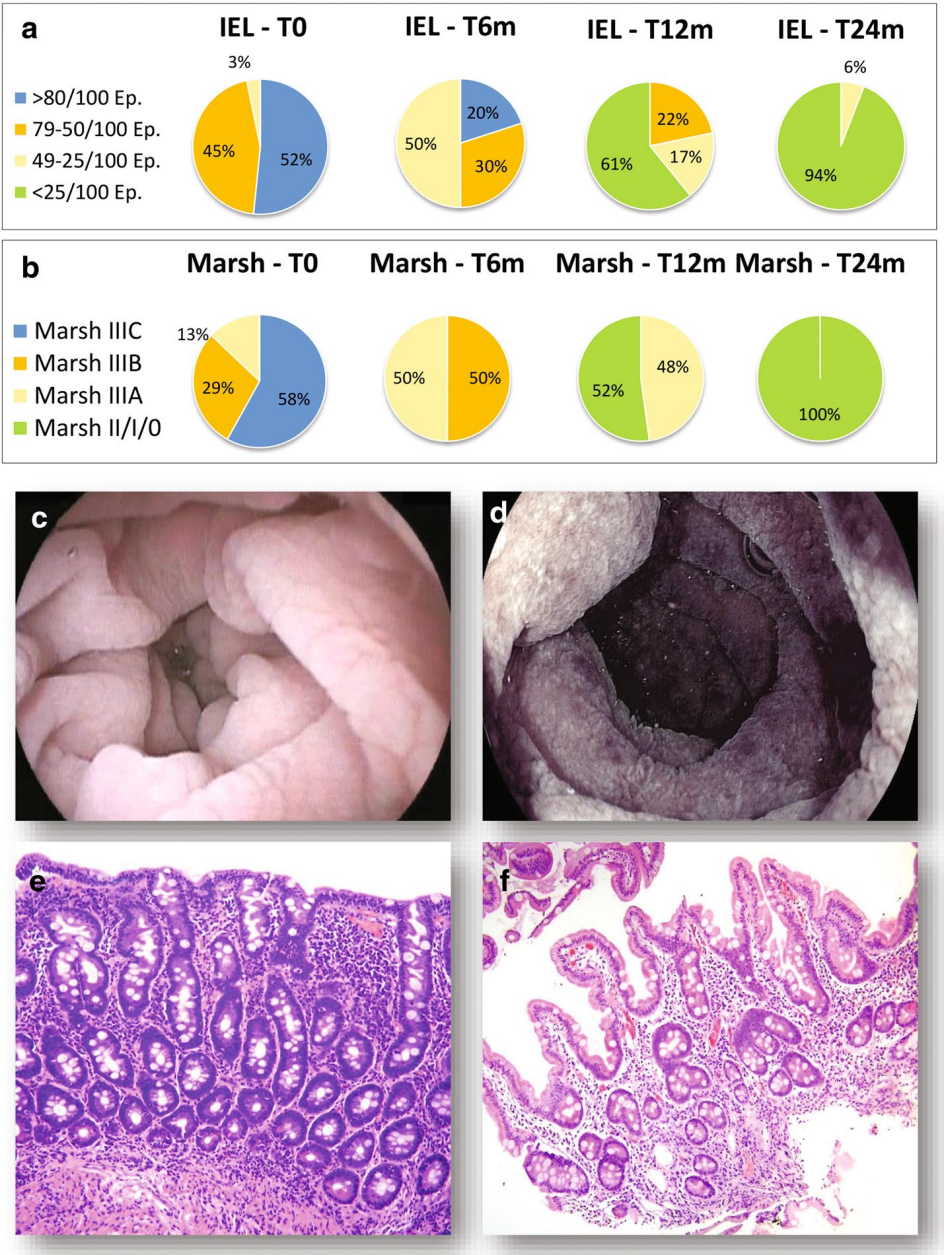
Longitudinal modifications of histological severity of duodenal damage in CD patients following GFD. **a** IEL: at baseline, 52% of CD patients presented with IEL >80/100 Epithelial cells (Ep.); 45% with IEL between 50 and 79/100 Ep. and the remaining 3% with IEL between 25 and 49/100 Ep. Upon GFD commencement, IEL progressively decreased. **b** Histological severity of duodenal damage evaluated according to the Marsh classification modified by Oberhuber (Marsh): at baseline, 58% of patients presented with a Marsh IIIc; 29% with Marsh IIIb and the remaining 13% with Marsh IIIa. Upon GFD commencement, all patients experienced a progressive villous regeneration. **c** and **d** depict representative CD patients’ endoscopic pictures (water immersion technique), while panels **e** and **f** correspond to their histological appearance (H&E staining). *T0* baseline, *T1m, T6m, T12m and T24m* 1, 6, 12 and 24 months following gluten removal from diet, respectively.

At enrolment, all CD patients showed high titers of atTG and EMA, sub-total or total villous atrophy (Marsh grade III) and abundant CD3^+^ IEL at histology (we assumed intraepithelial lymphocytosis to be present if >25 IEL/100 epithelial cells were observed) (Figure 2). All controls had normal duodenal aspect at endoscopy and normal histology at biopsy (Marsh 0). As expected, upon starting GFD, celiac patients experienced a progressive decrease in serum atTG and EMA levels and a gradual clinical improvement (data not shown). This was mirrored by progressive villous regeneration and IEL decrease at histology (Figure 2a). After 24 months of GFD, all patients were asymptomatic and their duodenal mucosa was macroscopically normal; at histology, none of the patients had villous atrophy and only 6% of them still showed a slight IEL increase (Marsh I) (Figure 2b).

**Figure 3:**
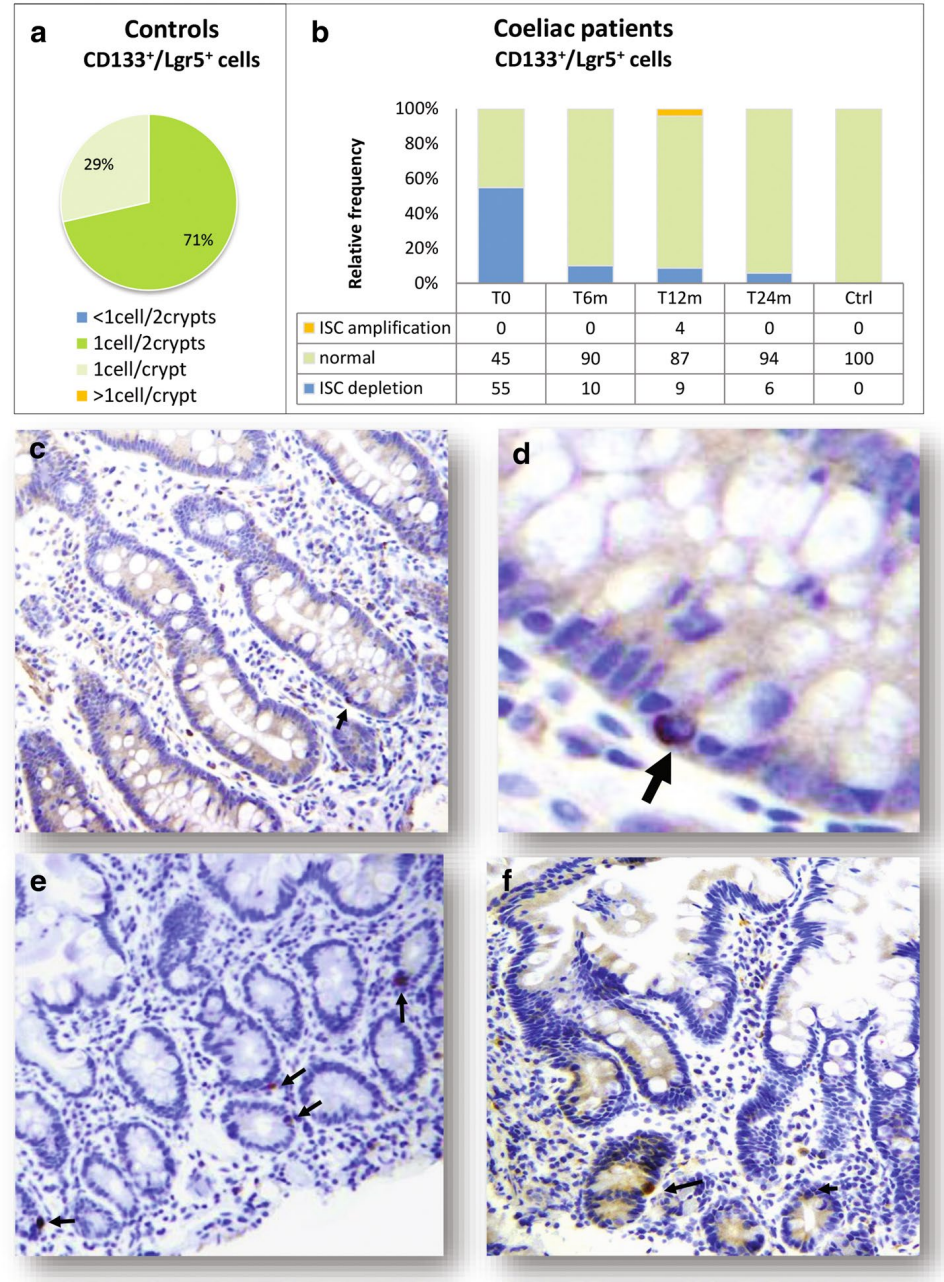
Putative intestinal stem cell density in controls and in CD patients. **a** 71% of non-coeliac subjects had one CD133^+^ epithelial cell every two crypts and 29% had one CD133^+^ cell per crypt; similar results were obtained for Lgr5^+^ intestinal cells. Therefore, we defined as “normal” a putative ISC density of 0.5–1 CD133^+^ or Lgr5^+^ cell/crypt. **b** At baseline, 55% of CD patients presented with reduced CD133^+^ and Lgr5^+^ intestinal cells (less than one every two crypts), whereas only 45% had a normal CD133/Lgr5^+^ representation. We defined as “ISC depletion” an ISC density <0.5 CD133^+^/Lgr5^+^ cell/crypt. Upon dietary intervention, the number of CD133^+^ and Lgr5^+^ epithelial cells significantly increased at 6 months and reached a peak at 12 months of GFD, when in some patients the putative ISC density was augmented versus controls. We defined a density >1 cell/crypt as “ISC amplification”. After 24 months of GFD, the CD133/Lgr5^+^ cell density was similar in patients and controls. Panels **c** through **f** show representative immunohistochemistry pictures for CD133^+^ putative ISC (*arrows*) in CD patients at baseline (**c**, higher magnification in **d**), at 12 months (**e**) and at 24 months (**f**) of GFD.

Regarding the putative ISC compartment (CD133^+^ and Lgr5^+^ intestinal cells), we firstly assessed its cell density at the basis of the crypts in the control population: 71% of non-coeliac subjects had one CD133^+^ epithelial cell every two crypts and 29% had one CD133^+^ cell per crypt; similar results were obtained for Lgr5^+^ intestinal cells. Therefore, we defined as “normal” a putative ISC density of 0.5–1 CD133^+^ or Lgr5^+^ cell/crypt (Figure 3a).

On the contrary, in 55% of active CD patients, CD133^+^ and Lgr5^+^ intestinal cells were less than one every two crypts, whereas only 45% had a normal CD133/Lgr5^+^ representation. We defined as “ISC depletion” an ISC density <0.5 CD133^+^/Lgr5^+^ cell/crypt. Such difference in putative ISC density between controls and active CD patients was statistically significant (*p* < 0.05). Upon dietary intervention, the number of CD133^+^ and Lgr5^+^ epithelial cells significantly increased at 6 months and reached a peak at 12 months of GFD, when in some patients the putative ISC density was augmented versus controls (*p* < 0.05). We defined a density >1 cell/crypt as “ISC amplification”. After 24 months of GFD, the CD133/Lgr5^+^ cell density was similar in patients and controls (*p* > 0.05) (Figure 3b–f).

## Discussion

The intestinal tract has developed mechanisms to cope with the harsh mechanical and chemical conditions to which it is exposed, via a highly regulated process of self-renewal and turnover [7]. According to the “unifying hypothesis”, all the differentiated epithelial cells of the gut derive from a single ISC compartment, which resides at the crypt base. From their niche, ISC generate transit-amplifying cells that migrate upwards and progressively lose their proliferative capability and become fully differentiated villous epithelial cells. In 1974, two distinct theories were formulated regarding the identity and location of the ISC: the “+4 position model”, postulating that ISC correspond to slowly cycling label retaining cells (LRC) in +4 position within the crypt; and the “stem cell zone model”, that identified the ISC with small and rapidly proliferating crypt base columnar cells (CBC) [8]. Since the 1970s, several experimental models have supported the concept of ISC; such studies have led to the hypothesis of an ISC hierarchy, organized in two main compartments (quiescent LRC and actively cycling CBC), progressively recruited as a result of various degrees of damage, in order to ensure an effective crypt regeneration [9–11]. Among the candidate markers for ISC, CD133 and Lgr5 are particularly promising. The glycoprotein CD133 (prominin-1) is expressed in immature HSC, as well as in other stem and progenitor cells including neural and embryonic stem cells [12, 13]. CD133 has been used as a colon cancer stem cell marker [14] and has also been suggested as a reliable marker for ISC [15]. The leucine-rich repeat–containing G-protein–coupled receptor-5 (Lgr5) is one of the target genes of the Wnt-pathway and it has recently been shown to specifically label stem cells in the mouse small intestine [16, 17]. Interestingly, lineage-tracing studies showed that some Lgr5^+^ cells co-express CD133 and these CD133^+^ cells can generate the entire intestinal epithelium [14, 18].

Besides resident ISC, circulating multipotent stem cells of bone marrow (BM) origin represent another potential source for intestinal regeneration. Indeed, over the last years, several studies have demonstrated that BM-derived HSC may participate in gut repair at various levels: by giving rise to ISC, providing supporting elements within the ISC niche, and releasing soluble factors that promote healing through immune modulation, inhibition of apoptosis, and stimulation of resident ISC [19–21].

A better knowledge of ISC function and dysregulation in chronic inflammatory bowel diseases might help to understand the pathophysiology of such disorders and might also offer new insights into the development of stem cell-based therapies [11].

In particular, to the best of our knowledge, there is only one published study about the potential role of circulating BM-derived stem cells in CD, by Mastrandrea and coworkers, who found an increased traffic of circulating CD34^+^ HSC in active CD patients versus healthy controls [22]. The authors postulated that this phenomenon was related to the prevalence of apoptotic versus survival programs, a defect shared by chronic inflammatory diseases; in such conditions, HSC might represent a supplementary ISC source.

Indeed, it has been demonstrated that in active CD the status of chronic inflammation worsens the epithelial layer damage, thus causing activation of NF-κB, which leads to a vicious cycle of aberrant immune response, mucosal inflammation, increased mucosal permeability and impairment of the regenerative potential of the intestinal epithelium. Moreover, it has been shown that gliadin-derived cytotoxic peptides can induce oxidative stress, rearrangement of actin cytoskeleton, impairment of epithelial tight-junction assembly, and deregulation of the epithelial homeostasis and this might further impair the ISC functions [23–25].

Regarding ISC modulation and deregulation in CD, it has been observed that ISC differentiation towards Paneth cells (PC) and goblet cells may be disturbed in active CD. Rubio et al., found that PC in active CD are replaced by lysozyme-producing mucus cells and speculated about an ISC reprogramming, as an antimicrobial adaptation to signals generated by pathogenic duodenal bacteria [26]. Capuano et al., observed a down-regulation of Notch pathway and KLF4 signals in CD patients and a reduction of the number of goblet cells in small intestine of children with active CD and in those on a GFD, compared to controls [27].

Our results confirm an increased traffic of CD133^+^CD34^+^ HSC in the peripheral blood of active coeliac patients. Additionally, we observed for the first time that the putative ISC compartment, represented by of CD133^+^/Lgr5^+^ crypt epithelial cells, is depleted in active CD; this result is in line with the above-mentioned theory of impairment of the intestinal regenerative potential in CD. Interestingly, we found that circulating HSC traffic significantly increases in the first week of gluten-free diet, suggesting that BM-derived stem cells might participate in the enteric repair process at the very beginning of the dietary intervention, when the local ISC niche is depleted. Afterwards, the circulating HSC progressively decrease, together with the immune-mediated intestinal damage, and the local ISC compartment expands, leading to villous regeneration and clinical remission.

Given the retrospective nature of our study, it is not possible to determine whether and to what extent the HSCs are contributing directly to the repopulation of depleted ISCs. This hypothesis should be tested in animal models of CD, for instance upon sex-mismatched HSC transplantation [28].

Following a similar approach, BM contribution to the intestinal regeneration has been demonstrated in experimental models of inflammatory bowel disease (IBD); this evidence supported the rationale for the pharmacological mobilization of HSCs in the treatment of IBD [29, 30].

Finally, the dramatic increase in circulating HSC following initiation of GFD is worth speculation. We could hypothesize that the circulating BM-derived stem cells are migrating to sites of inflammation and being consumed in the lesions, and once the inflammation is reduced, their numbers increase in the circulation. Alternatively, it is possible that gluten removal induces the injured gut to release factors involved in HSC mobilization and homing, such as G-CSF and SDF-1 [31]. Experimental studies to assess intestinal gene expression modifications upon gluten removal would be of help to clarify the mechanisms underlying such HSC longitudinal modifications following GFD in CD.

## Conclusions

The pathogenic interactions among genetic, immunological, and environmental factors in CD have been extensively investigated. On the contrary, little is known about ISC modulation and deregulation during the course of the disease. In the present study, we observed that the putative ISC compartment is depleted in active CD, likely due to a status of chronic inflammation, that leads to the impairment of the regenerative potential of the intestinal epithelium. Removing the inflammation-inducing factor (gluten) allowed for repopulation of the ISC compartment to occur. Circulating HSCs might actively contribute to this repopulation process, as suggested by the significant increase of HSC traffic at the very beginning of the dietary intervention. Afterwards, the circulating HSCs progressively decreased and the local ISC compartment expanded, leading to villous regeneration.

This study sheds some light on the dynamics of intestinal regeneration following GFD in coeliac patients and offers novel insights for the development of HSC-based treatments against CD. Further studies are required to dissect the molecular mechanisms underlying ISC impairment in active CD and their expansion upon gluten removal from the diet, and also to clarify the role of BM-derived stem cells in intestinal regeneration.

## Abbreviations

ISC: intestinal stem cells
HSC: hematopoietic stem cells
CD: coeliac disease
GFD: gluten-free diet
IEL: intraepithelial lymphoid infiltrate
atTG: anti-tissue transglutaminase antibodies
EMA: anti-endomysial antibodies
PE: phycoerythrin
FITC: fluorescein isothiocyanate
WBC: white blood cells
H&E: hematoxylin-eosin
LRC: label retaining cells
CBC: crypt base columnar cells
BM: bone marrow
PC: Paneth cells
IBD: inflammatory bowel diseases
